# Supplementary material for: Utilization of a Low-Cost Sensor Array for Mobile Methane Monitoring
Source: Sensors (Basel). 2024 Jan 14;24(2):519. doi: 10.3390/s24020519 (PMC10820073; doi:10.3390/s24020519)
Supplement: Supplementary file 1 [file sensors-24-00519-s001.zip › Supplementary_Figures_Final.pdf]

1. Supplementary Figures

List of Figures

S1 XPOD low-cost methane sensor configuration. 2
S2 Inlet setup atop monitoring vehicle. 2
S3 Oil and gas storage and flaring facility on the Eastern plains of Colorado 3
S4 Oil and gas facility on the Eastern plains of Colorado. 3
S5 Pre- and post-binning reference methane distribution. 4
S6 Sample ANN architecture employed in model fitting. 4
S7 Time series plot comparison during data collection on August 30th, 2023 5

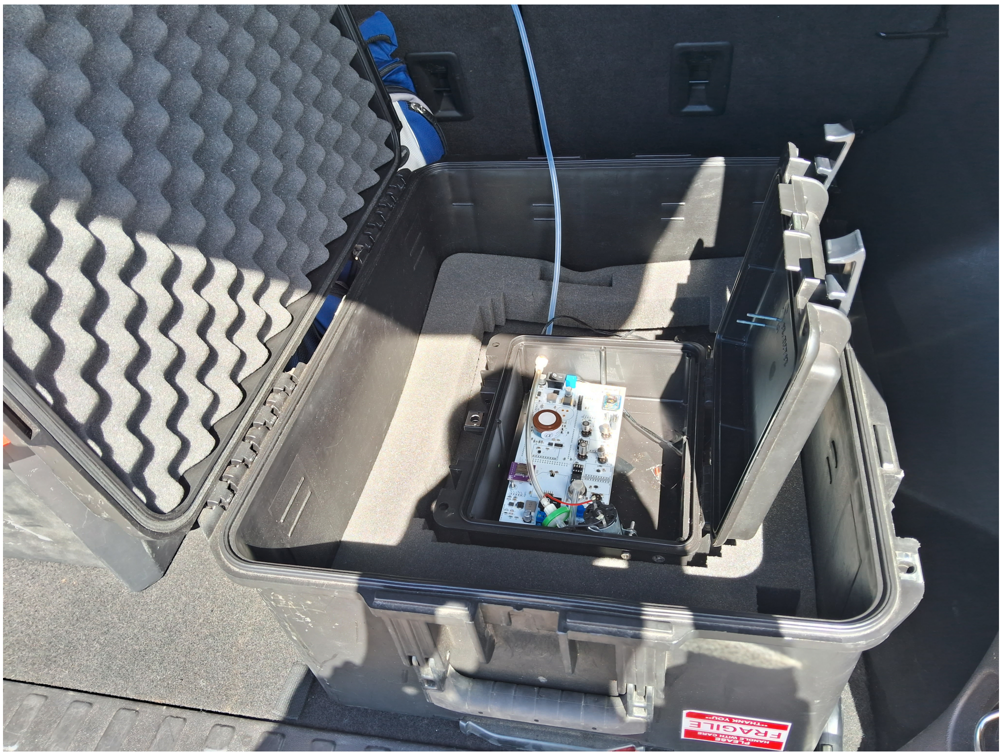

Figure S1: XPOD low-cost methane sensor configuration.

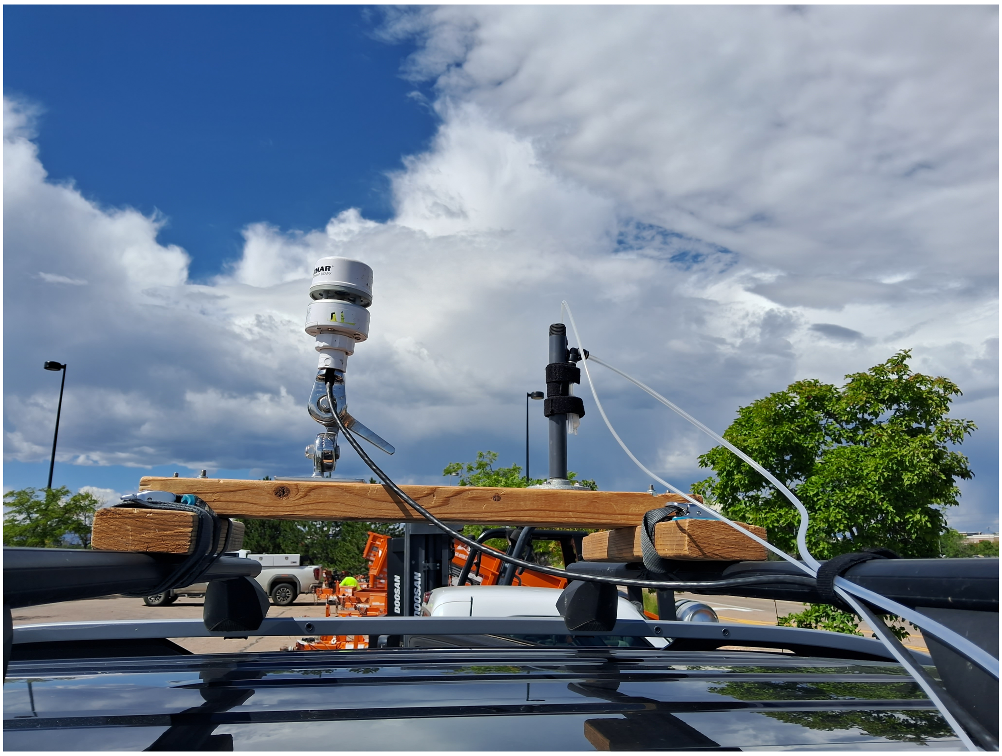

Figure S2: Inlet setup atop monitoring vehicle.

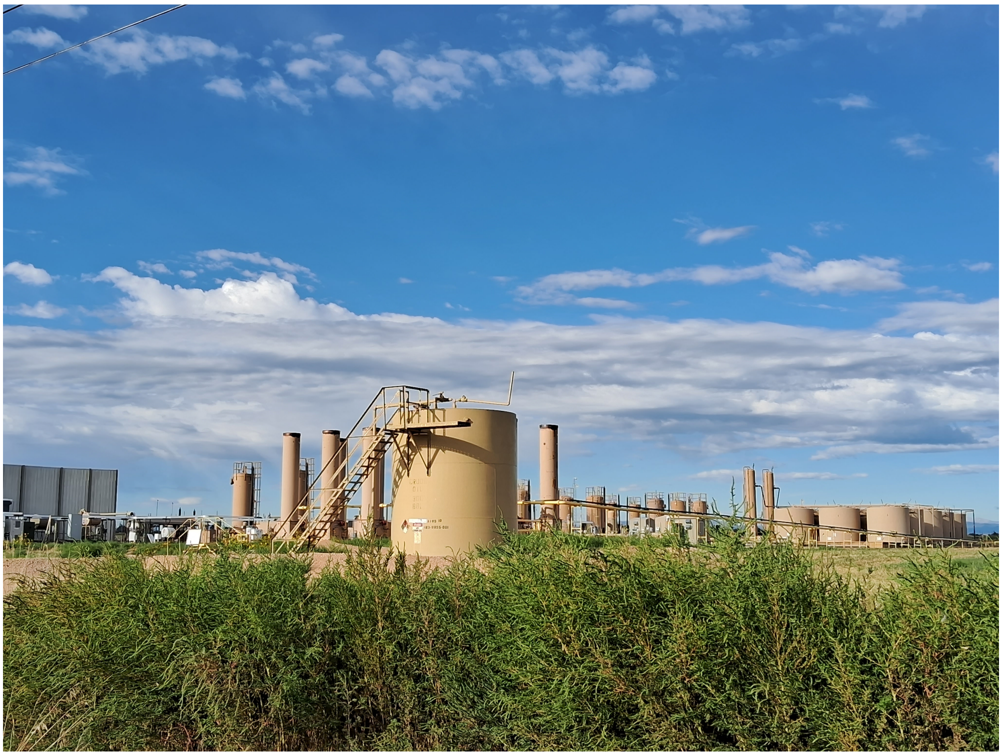

Figure S3: Oil and gas storage and flaring facility on the Eastern plains of Colorado

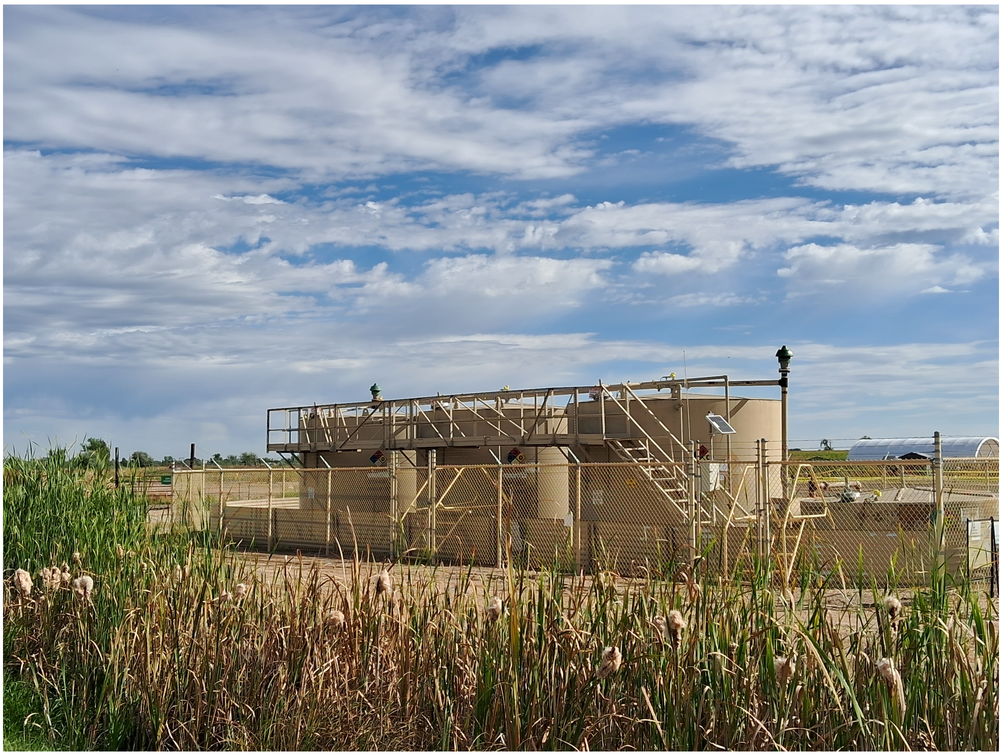

Figure S4: Oil and gas facility on the Eastern plains of Colorado.

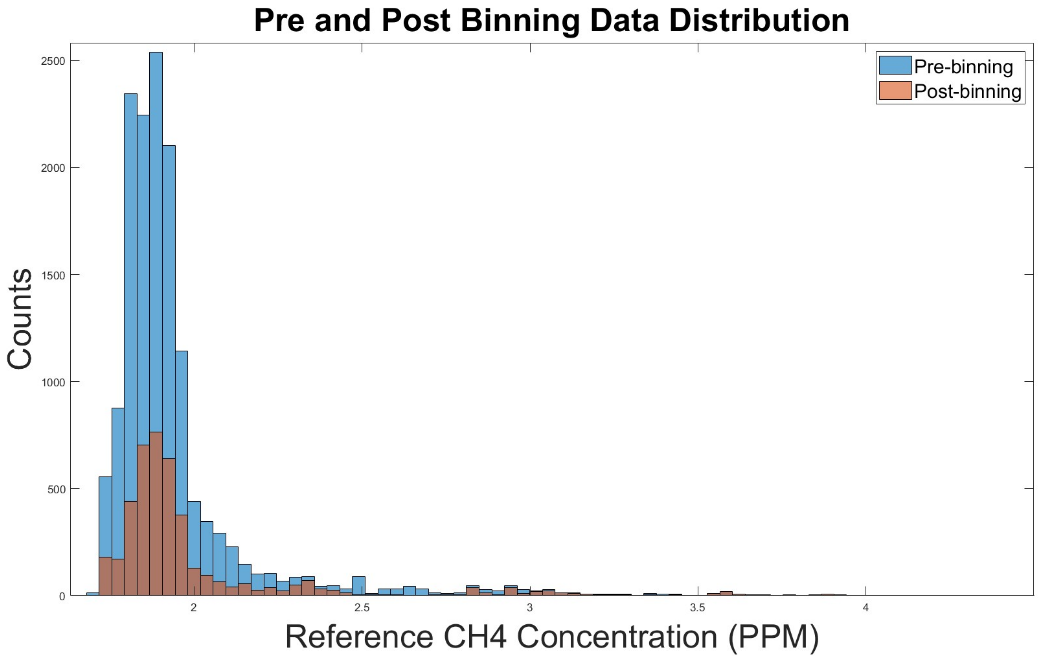

Figure S5: Pre- and post-binning reference methane distribution. Binning data reduced the overall number of data points in our sample set but resampled the overall distribution so that baseline concentrations of CH_4 were not oversampled for model fitting.

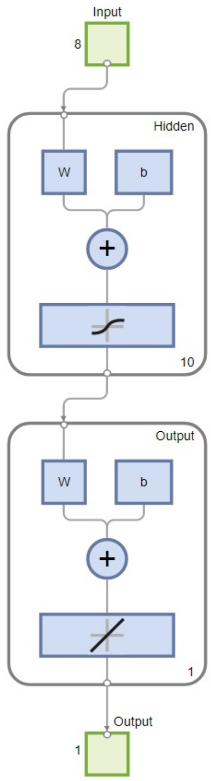

Figure S6: Sample ANN architecture employed in model fitting.

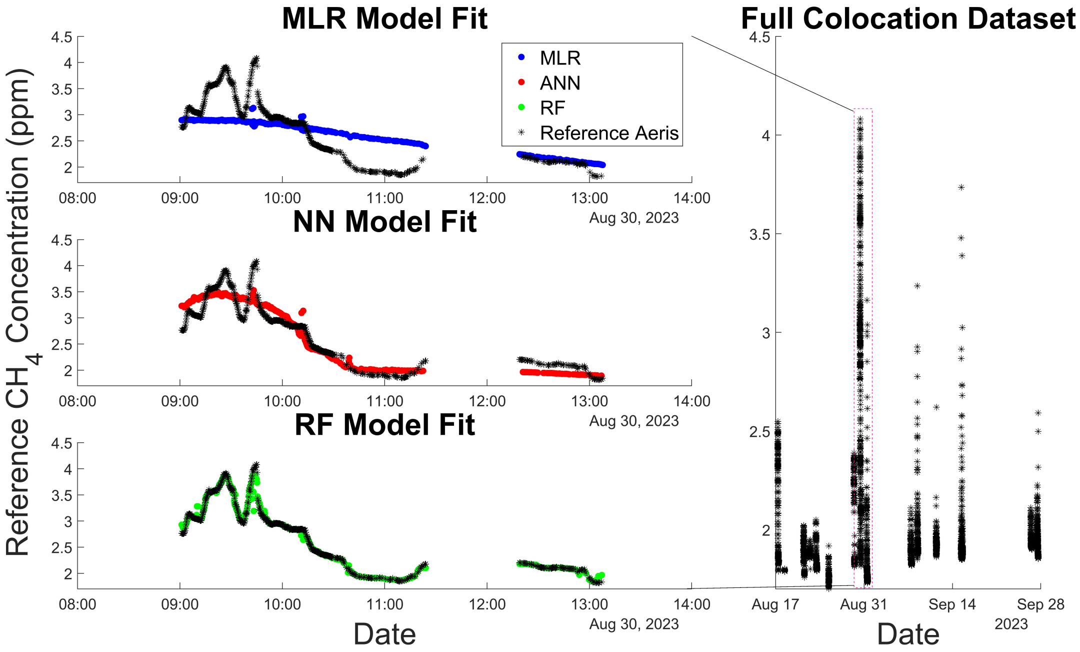

Figure S7: Time series plot comparison during data collection on August 30th, 2023. Note that the MLR model is able to capture the general trend and fits baseline data well. The neural network does an improved job of fitting the local variation in CH_4 but misses peaks. The RF model is able to fit both short-term CH_4 peaks and baseline CH_4 values.
